# Selenium Regulates Antioxidant, Photosynthesis, and Cell Permeability in Plants under Various Abiotic Stresses: A Review

**DOI:** 10.3390/plants12010044

**Published:** 2022-12-22

**Authors:** Haodong Liu, Chunmei Xiao, Tianci Qiu, Jie Deng, Hua Cheng, Xin Cong, Shuiyuan Cheng, Shen Rao, Yue Zhang

**Affiliations:** 1 School of Modern Industry for Selenium Science and Engineering, National R&D Center for Se-Rich Agricultural Products Processing Technology, Wuhan Polytechnic University, Wuhan 430023, China; 2 Enshi Se-Run Material Engineering Technology Co., Ltd., Enshi 445000, China

**Keywords:** abiotic stress, antioxidant, cell membrane, plant, selenium

## Abstract

Plant growth is affected by various abiotic stresses, including water, temperature, light, salt, and heavy metals. Selenium (Se) is not an essential nutrient for plants but plays important roles in alleviating the abiotic stresses suffered by plants. This article summarizes the Se uptake and metabolic processes in plants and the functions of Se in response to water, temperature, light, salt, and heavy metal stresses in plants. Se promotes the uptake of beneficial substances, maintains the stability of plasma membranes, and enhances the activity of various antioxidant enzymes, thus alleviating adverse effects in plants under abiotic stresses. Future research directions on the relationship between Se and abiotic stresses in plants are proposed. This article will further deepen our understanding of the relationship between Se and plants.

## 1. Introduction

The environment is complex and varied for plants. Adverse conditions can cause biotic and abiotic stresses in plants. Biotic stresses include insect pests, diseases, and weeds, whereas abiotic stresses are classified as water, temperature, light, salinity, and heavy metals. Abiotic stresses are the main environmental factors that inhibit plant growth and reduce crop productivity and quality. Abiotic stresses cause significant molecular and physiological changes in plants, including rapid transcriptional and metabolic adjustments, osmotic potential regulation, reduction in leaf expansion pressure, and ultimately lead to the slowdown or cessation of plant growth [1,2]. Abiotic stress tolerance is a key factor in determining crop productivity. Thus, strategies to improve plant tolerance to abiotic stresses have attracted increasing interest. These strategies include applying plant hormones [3] and altering cultivation practices [4].

Selenium (Se) is a non-metallic element of the oxygen group. It is an essential trace element for humans and animals and plays important roles in their health [5]. Recent studies have shown that Se is not an essential nutrient for plants but can be taken up and metabolized by plants. According to the Se concentration of plant shoots under natural conditions, plants can be classified into non-accumulators (<100 mg kg^−1^ Se DW) [6], secondary accumulators (100–1000 mg kg^−1^ Se DW), and hyperaccumulators (>1000 mg kg^−1^ Se DW). Although the ability of different plants to absorb and enrich Se varies greatly, low concentrations of Se are beneficial to plant growth, whereas high concentrations can be toxic. For example, Khaliq et al. [7] soaked rice seeds with a low concentration of Se (15–60 µmol L^−1^) solution and found that this treatment significantly promotes the emergence rate of rice seeds and growth of rice seedlings. Moulick et al. [8] soaked rice seeds with 2.89–8.67 µmol L^−1^ Na_2_SeO_3_ solution and found that this treatment significantly increases the chlorophyll content, height, spike length, and grain weight of rice plants, which ultimately improve the yield and quality of rice. By contrast, high concentrations of Se can be harmful to plant growth [9]. Treatment of *Cardamine violifolia* with 80 µmol L^−1^ sodium selenate can significantly reduce the chlorophyll content and biomass, and the plants are under the symptoms of poisoning [10]. The high Se accumulation caused by 200–400 µmol L^−1^ selenate might be beyond the Se tolerance of the plant and generate Se stress, seriously inhibiting the growth and causing abnormalities in the nutrition synthesis and metabolism of *C. violifolia* [11].

Recently, many studies have found that appropriate concentrations of Se can enhance the abiotic stress tolerance of plants. Djanaguiraman et al. [12] found that Se application can reduce the membrane damage of sorghum cells by enhancing antioxidant defense, thus protecting cell integrity, improving heat tolerance, and reducing yield losses in sorghum under high-temperature stress. Hasanuzzaman et al. [13] also revealed that Se application enhances the antioxidant defense and methylglyoxal detoxification systems in plants, thereby increasing tolerance to drought-induced oxidative damage. Similarly, Se improves the nutrient uptake, amino acid metabolism, and antioxidant defense system in kale oilseed rape under chromium (Cr) stress, thus reducing Cr toxicity [14]. This review mainly summarizes the recent studies on the regulatory roles of Se in the photosynthetic characteristics, antioxidant enzyme activity, and cell permeability in plants under abiotic stresses. This article will help us understand the functions of Se in responding to abiotic stresses in plants and the relationship between Se and plants.

## 2. Se Metabolism in Plants

### 2.1. Se Uptake by Plants

The two main forms of bioavailable Se in soils are selenate and selenite. Selenate is mainly found in alkaline and oxidized soils, whereas selenite mainly exists in acidic and reduced soils [15]. Plants also can take up organic Se, such as seleno-amino acids, but plants cannot absorb elemental or metal-selenide compound complexes [6,16].

**Figure 1:**
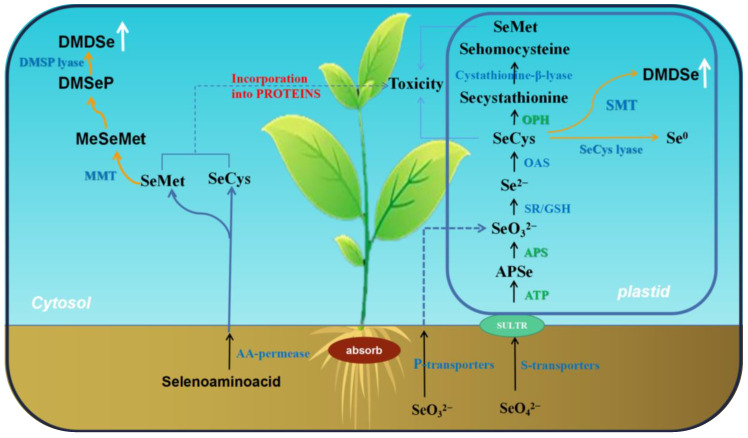
Schematic diagram of Se assimilation and metabolism in plant cells. SULTR, sulfate/selenate cotransporter; APSe, adenosine phosphoselenate; APS, adenosine phosphosulfate; SR, Sulfite Reductase; GSH, glutathione; SAT, serine acetyltransferase; OAS, O-acetylserine; (Se) Cys, (seleno)cysteine; OPH, O-phosphohomoserine; (Se)Met, (seleno) methionine; MMT, methylmethionine methyltransferase; DMSeP, dimethylselenoproprionate; DM(D)Se, dimethyl(di)selenide (volatile); SMT, selenocysteine methyltransferase.

The uptake and assimilation pathway of selenate by plants has been well uncovered currently (Figure 1). Selenate is taken up by the sulfate uptake transporter system in plants [17]. Sulfate transporters (SULTR) in plants have four major groups. The first group (SULTR1) is composed of high-affinity sulfate transporters, including SULTR1;1 [18] and SULTR1;2, which are located in the root system and are the most studied transporters. They are responsible for the uptake of selenate from the soil, but evidence shows that SULTR1;2 is the predominant selenate uptake protein, rather than SULTR1;1. The second group (SULTR2) is composed of low-affinity sulphate transporters and located in various tissues of plants, including the two isoforms SULTR2;1 and SULTR2;2. They are located in the leaves and roots and play important roles in the entry of selenate into the vascular system [19]. The third group (SULTR3) is found only in the leaves. SULTR3;1 is located in chloroplasts. The biosynthesis reduction of this transporter leads to a decrease in sulfate content in chloroplasts [20]. The fourth group (SULTR4) is found in the vacuole membrane. This group contains two isoforms, namely, SULTR4;1 and SULTR4;2. They are associated with sulfate transfer in the vacuole [21].

Different from selenate, the uptake of selenite in plants may be attributed to phosphate transporters and water channel proteins [22,23,24]. A recent study has shown that Se application on rice can significantly upregulate the expression of phosphate transporter (OsPT2), implying that it may play a key role in the uptake of selenite in rice [23,25,26]. Plants can also take up organic Se via amino acid permeases. Two common types of organic Se are selenocysteine (SeCys) and selenomethionine (SeMet) [16]. Kikkert and Berkelaar found that the uptake of SeCys and SeMet is 20- and 40-fold faster than that of selenate in spring canola and 2- and 100-fold faster in wheat [16]. This difference in uptake is mainly related to the differences in Se species and their concentrations.

### 2.2. Se Assimilation by Plants

Plants metabolize Se via the sulfur metabolic system because of the chemical similarity of the two elements [27]. Similar to that of sulfur, the assimilation of Se occurs in the cytosol and plastids of plant leaf cells [28]. Selenate is translocated into the leaves after being taken up by the roots. Selenate is firstly activated by adenosine triphosphate sulfurase to form adenosine 5′-phosphoselenate and continuously reduced by adenosine 5′-phosphosulfate reductase to generate selenite [29,30]. Selenite can be further converted into selenide. The reduction of sulfite into sulfide is achieved by the action of sulfite reductase (SR) during sulfate metabolism [31]. The reduction from selenite to selenide may also be achieved by the action of SR. Subsequently, selenide is further transformed into SeCys under the catalysis of cysteine synthase [15,32]. Plants cannot distinguish between SeCys and Cys; thus, SeCys can displace Cys and be non-specifically incorporated into proteins, resulting in protein dysfunction and causing plant toxicity [33]. Some plants retain the capacity to process SeCys into non-toxic forms, thus avoiding Se toxicity. Part of SeCys is broken down to elemental Se by the mediation of SeCys lyase. Elemental Se can be excreted by plant cells, thus declining the Se concentration in the plant body. The other part of SeCys is converted into selenocystathionine by cystathionine-γ-synthase and then into selenohomocysteine by cystathionine-β-lyase. Selenohomocysteine would be transformed into SeMet under the catalysis of Met synthase [34]. Plants also can process SeCys and SeMet into non-protein amino acids. SeCys and SeMet can be converted into methyl SeCys (MeSeCys) and methyl SeMet (MeSeMet) under the mediation of SeCys methyltransferase and S-adenosyl-L-Met:Met-S-methyltransferase, respectively. This process can reduce the misincorporation of SeCys and SeMet into proteins [15]. MeSeCys and MeSeMet are further converted into volatile dimethyl diselenide and dimethyl selenide. The two volatile compounds would be excreted from plant cells, thereby reducing the Se concentration in plants and reducing Se damage to plants [35,36].

## 3. Se and Stresses in Plants

### 3.1. Se and Drought

Water stress on plants can be divided into two types, drought and flooding, both of which are very detrimental to plant growth. Among the various environmental stresses, drought is the most damaging to plants. Drought can reduce chlorophyll content [37] and cause stunted shoot growth but stimulate root growth [38]. The correlation between Se and flooding stress in plants remains unclear to date. However, studies have demonstrated that Se can effectively alleviate drought stress in crops [39]. The mechanism involved in this effect can be categorized into the following aspects.

Se increases the activity of antioxidant enzymes in plants and improves the non-enzymatic breakdown of superoxide radicals [40], thereby reducing the accumulation of excess reactive oxygen species (ROS) and mitigating oxidative damage to cell membranes. ROS play important roles in plant resistance to abiotic stresses [41]. Plants subjected to drought produce large amounts of ROS, including superoxide anions, hydrogen peroxide, hydroxyl radicals, singlet oxygen, and methyl and lipid peroxide radicals [42]. The accumulation of ROS in plant cells can cause varying degrees of oxidative damage to biological membrane proteins and DNA and further disrupt the respiration and photosynthesis of plant cells [43]. Suitable concentrations of Se can increase the activities of antioxidant enzymes or the contents of other antioxidant substances, thus accelerating the scavenging of intracellular free radicals [44]. ROS elimination helps prevent oxidative stress and enhance the antioxidant capacity of plants. Cucumber seedlings grown in nutrient solution with 1 and 5 µmol L^−1^ have higher drought tolerance capacity than normal ones because the former have increased superoxide dismutase (SOD), peroxidase (POX), ascorbate peroxidase (APX), and catalase (CAT) activities and reduced ROS accumulation in the root system [45]. Se also effectively reduces drought stress in olives. Spraying of olive leaves with 50 and 150 mg Se L^−1^ can significantly enhance the activities of APX, CAT, and glutathione peroxidase (GPOX) and the content of malondialdehyde (MDA), thus effectively scavenging excess ROS and protecting cells from oxidative damage [46].

Se enhances water uptake and the relative water content in plants. Relative water content is an indicator of plant water status and reflects the balance between water availability and the transpiration rate of leaf tissues [47]. The relative intracellular water content is significantly reduced when plants are under drought and inhibited nutrient uptake [48], which can lead to plant death in the long term. Exogenous Se can increase the relative water content of plant cells in crops, such as sorghum [49], spring barley [50], and canola [51], consequently reducing the stress-generated damage and improving the drought tolerance of plants. The underlying mechanism is that Se promotes water uptake by the root system of plants under drought conditions. However, the details underlying the mechanism remain to be revealed. Se can possibly enhance the vigor of the root system to absorb water under drought conditions.

Se protects chlorophyll and maintains photosynthesis. Drought stress causes water deficiency in plants, closing of leaf stomata, and induction of stomatal conductance [52], which would further inhibit the supply of CO_2_ and reduce the rate of CO_2_ assimilation [53]. Therefore, long-term drought would eventually damage chlorophyll and prohibit photosynthesis in plants [50]. Se can effectively mitigate this damage to plants. Application of a 30 g/ha solution of Na_2_SeO_4_ to the leaves can significantly enhance stomatal conductance in spring barley cells under drought stress, thus enhancing photosynthesis [50]. Drought stress in rice can significantly reduce photosynthesis. Application of soil Se at a concentration of 0.5 mg kg^−1^ increases chlorophyll index, CO_2_ assimilation efficiency, and net photosynthesis in rice [44]. Application of low concentrations of Na_2_SeO_4_ to canola under drought stress can significantly increase chlorophyll a and chlorophyll b contents, which enhance photosynthetic efficiency [51]. This phenomenon may be ascribed to that Se can reduce the accumulation of ROS in plants subjected to drought [42]. However, the exact mechanisms underlying this effect remains unclear.

Interestingly, Zhou et al. [54] found that flooded irrigation treatment increases the soil soluble Se concentration, and the Se in soil solution is present in the form of selenite and selenomethionine Se oxide. On the one hand, enhancement in the concentrations of soluble Se in the soil through flooding irrigation can significantly promote Se levels in grain and straw rice. On the other hand, Se at a relatively higher level is effective in mitigating the adverse effects of flooding in plants. Thus, irrigation practices and Se application can form a virtuous circle for plant growth.

### 3.2. Se and Temperature Stress

**Figure 2:**
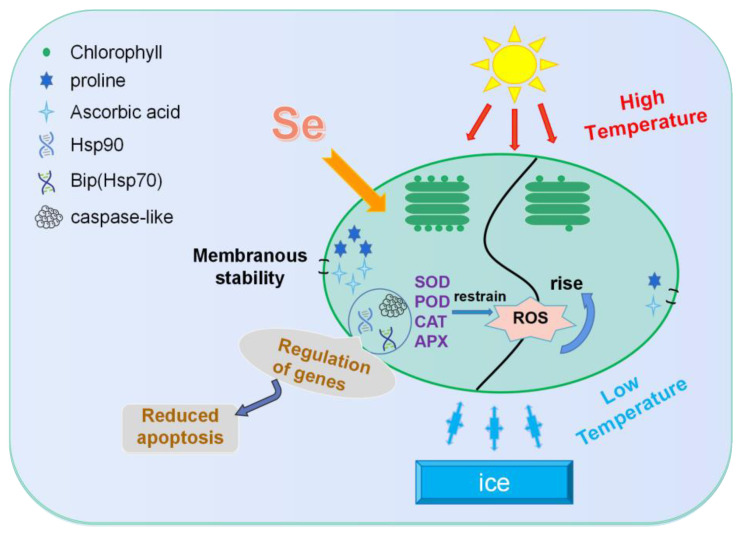
Patterns of Se mitigating temperature stress in plants. The ellipse indicates plant leaf cells under high and low-temperature stress, the left half indicates cells treated with Se.

Temperature is important for plant growth. The biochemical reactions and physiological activities in plant cells are dependent on suitable temperature. Temperature stress is an important abiotic stress for plant growth because it causes serious damage to plants, including dysregulation in photosynthetic processes, disturbance in enzyme activity, and metabolic disorders [55,56]. High-temperature stress causes premature leaf senescence, resulting in loss of chlorophyll, aggravation in membrane damage, and a decline in photosynthetic capacity [57]. By contrast, low-temperature stress inhibits chlorophyll biosynthesis and antioxidant enzyme activity but increases ROS production and cell membrane damage [58,59,60]. Therefore, high and low temperatures can restrain plant growth, even leading to plant death in severe cases. Se can effectively regulate high or low-temperature stress in plants. This effect is attributed to the following mechanisms (Figure 2).

(i) Se protects chlorophyll and helps maintain photosynthesis. High-temperature stress accelerates the degradation of chlorophyll [61], increases photorespiration, reduces ribulose-1, 5-bisphosphate carboxylase/oxygenase (RuBisco) activity, and interferes with the electron transport chain of the photosynthetic system in plants [62]. Se can protect chlorophyll and mitigate temperature stress-induced damage to photosynthesis [63]. Seliem et al. [64] found that Se nanoparticles (SeNPs) promote photosynthetic processes and increase the total chlorophyll content of different varieties of *Chrysanthemum morifolium* Ramat under heat stress at 40 °C. Similarly, in strawberry seedlings under low-temperature stress, the stomatal conductance is decreased and the concentration of intercellular carbon dioxide is increased. Spraying leaves with Se remarkably maintains the homeostasis of stomatal conductance and intercellular carbon dioxide in strawberry leaves, thus effectively repairing the photosynthesis and mitigating the damage of low temperature on strawberry plants [65]. Se can also contribute to the stabilization of iron-sulfur clusters in the photosystem and regulation of the flow of electrons in chlorophyll cysts at the subcellular level, thus enhancing the quantum yield of light response [42]. Therefore, Se helps maintain photosynthesis in crops under temperature stress.

(ii) Se improves the antioxidant capacity and alleviates the oxidative damage of plants under low temperatures. Wheat seeds soaked with 26.45 µmol L^−1^ sodium selenate and germinated at a low temperature (3 or 5 °C) show enhanced CAT and polyphenol oxidase activities and reduced ROS concentration [66], thus protecting the cell membrane. Wheat seedlings treated with Se under low-temperature stress also significantly enhance the activities of POD and APX [67]. A low concentration of sodium selenite solution (28.90 µmol L^−1^) significantly increases the activities of SOD, CAT, and POD in strawberry seedlings under low-temperature stress, which effectively alleviate oxidative damage in strawberry seedlings [65]. Low temperature and heat stress can be regulated by Se through changing the activities of antioxidant enzymes. In cotton under high-temperature stress (35/22 °C, day/night), Se enhances the activities of GPOX and CAT, thus increasing the heat tolerance of cotton [68]. However, a Se-induced increase in the activities of low-molecular-weight non-enzymatic antioxidants, such as glutathione (GSH), also contributes to the enhancement of heat tolerance in cotton [68].

Furthermore, Se may facilitate the dissipation of excess energy from photosystem ii reaction centers in plants under heat stress, thus slowing down ROS synthesis and effectively mitigating oxidative damage in plants [69].

(iii) Se enhances osmotic protection in plants. Proline and soluble sugars act as osmotic protectants and regulate cellular metabolic activities [70]. A high level of free proline is one of the factors enhancing cold tolerance in plants under low-temperature stress [71]. Soluble sugars act as osmotic protectants and important metabolic substrates and play a dynamic role in controlling numerous procedures in plant development [72].

Se can increase proline content in plants possibly by affecting the activities of proline synthesis-related enzymes [63,73]. Sorghum seeds exposed to low concentrations of selenate (15.87 and 31.75 µmol L^−1^) show a significant increase in proline and soluble sugar contents when germinated under low temperatures (4 °C or 8 °C) [67]. Similar phenomena were observed in potatoes and cucumber. Spraying 126.6 µmol L^−1^ SeNPs on the leaves of *Coriandrum sativum* can significantly increase it soluble sugar content by 1.5 times [74]. These studies suggest that appropriate concentrations of Se effectively increase the accumulation of proline and soluble sugar and enhance cold resistance in plants under low-temperature stress.

(iv) Se regulates the expression of genes associated with temperature stress. Tobacco cell cultures treated with selenate show lower expression levels of some stress-related protein genes, such as the heat-stimulated protein Hsp90, BiP (Hsp70 family), 14-3-3s, and cytochrome c, when they are under high-temperature stress (5 min, 50 °C). HSP90 and HSP70 function in organelle-specific protein sorting [75,76] and ubiquitin-mediated proteasomal degradation [77] and are related to thermal resistance in plants [78]. Therefore, the regulation of these genes by Se would contribute to protect plants from heat stress.

In summary, appropriate concentrations of Se are beneficial for protecting the physiological and biochemical activities of plants, thus effectively alleviating the negative effects of adverse temperatures. High and low temperatures are important abiotic stresses that affect crop production worldwide. Methods to mitigate the effects of adverse temperature on crop yield and quality are difficult to achieve. Application of Se may be a viable strategy to ameliorate the adverse effects of temperature stress on crops.

### 3.3. Se and Light Stress

Light is the only source of energy for compound accumulation and plays an important role in plant growth, physiology, biochemistry, and morphological establishment in plants [79]. Plants cannot photosynthesize adequately under low light, leading to a deficiency in energy and inhibiting their growth [80]. Strong light can damage the chloroplasts and weaken photosynthesis [81]. Studies have demonstrated that low concentrations of Se can protect cystoid membranes, maintain the stability of cystoids and chloroplast stroma, significantly increase the content of beneficial elements directly related to the structural function of chloroplasts in plant leaves, and aid in the recovery of membrane structure under low or strong light stress [75,82].

Se alleviates chloroplast damage by regulating antioxidant substances when plants are exposed to strong and low light. Potato plants exposed to 600 mmol m^−2^ s^−1^ of strong light show remarkable alteration in transcriptive levels of chloroplast CuZnSOD and GPX when they are treated with low concentrations of Se [83]. This result suggests that Se facilitates the activation of protection mechanisms when plants are exposed to intense light. Elevation in the response to oxidative stress induced by Se enhances the stability of photosynthetic pigments and promotes the recovery of chlorophyll in plants after light stress, even making the chlorophyll content reach the initial level in plants before light stress [84].

Ultraviolet (UV) light is another form of light stress aside from low or strong light. Recent studies have highlighted UV as a regulator of plant growth and development rather than as a destructive stressor [85]. High UV intensity can decrease photosynthetic pigment levels, respiratory potential, Ca^+^ concentration, and leaf thickness [86]. Se effectively mitigates the effects of UV radiation on plants. Pumpkin plants grown in the field are sensitive to UV-B radiation. UV-B negatively affects the electron flow at the end of the electron transport system and impairs the flow of electrons in the respiratory chain, thus decreasing the yield [87]. Foliar spraying of Se counteracts this effect, significantly increasing the fruit yield of plants exposed to UV-B radiation [87]. However, the underlying mechanism remains unclear. Evidence suggests that Se can enhance the accumulation of antioxidant substances, increase the activity of antioxidant enzymes, and reduce the accumulation of ROS in plants under UV stress. Wheatgrasses germinated from Se-rich wheat grains exhibit increased total flavonoid and phenolic contents than non-seleniferous ones when they are exposed to UV-B stress [88]. The former also shows a higher scavenging rate of DPPH radicals and activities of SOD and CAT but lower lipid peroxidation [88]. Treatment with Na_2_SeO_3_ solutions (28.90, 57.80, and 115.6 µmol L^−1^) also increases the isoflavone content and inhibition of NO production in soybean under UV stress [89]. The antioxidant activity of soybean cells is thus increased. Aside from increasing antioxidant capacity, Se can promote the accumulation of UV-absorbing compounds. Golob et al. [90] found that foliar spraying with 52.91 µmol L^−1^ sodium selenate significantly increases the content of some UV-absorbing compounds, such as Si and Ca, in wheat seedlings under UV radiation [91]. It can also increase light reflectance and reduce transmittance, thus enhancing the protection of wheat under UV stress [90].

### 3.4. Se and Salt Stress

**Figure 3:**
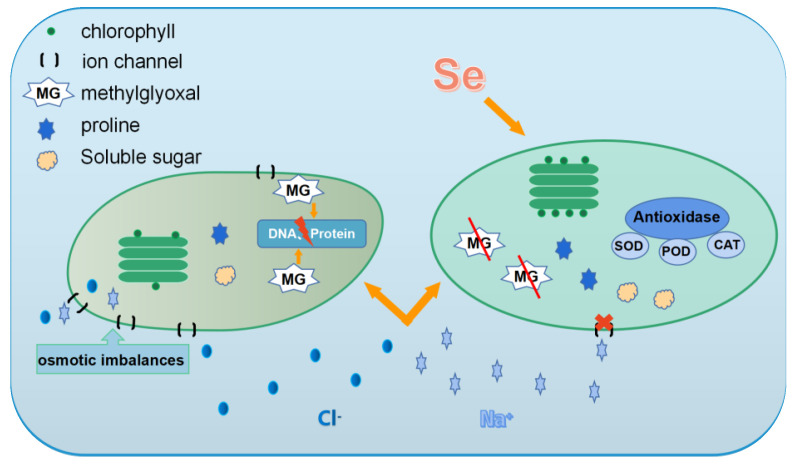
Patterns of Se mitigating salt stress in plants. The left shows plant cells under Na^+^ salt stress. The right shows a cell treated with Se under salt stress.

High salt levels in the soil cause salt stress in plants. Salt stress can induce nutritional imbalance, water deficit, oxidative stress, and disruption of cellular ion homeostasis in plants [92]. Plants can be generally classified into two major types according to salinity tolerance, namely, halophytes (salt-tolerant) and glycophytes (salt-sensitive). Glycophytic plants can tolerate relatively low salt concentrations ranging from 50 mM to 250 mM NaCl, whereas halophytic plants are adapted to natural conditions of high salt (approximately 500–1000 mM NaCl) in the soil [93]. The Food and Agriculture Organization of the United Nations states that salt stress poses a serious threat to more than 6% of the land [94]. Salt stress reduces the uptake of water and nutrients, increases the osmotic potential of plant cells [95], inhibits plant photosynthesis, and alters plant metabolism and physiology, thereby inhibiting plant growth and reducing yields [96]. For instance, when Na^+^ and Cl^-^ are taken up in large quantities by the root system, metabolic impairment and photosynthetic efficiency reduction would occur [97,98]. Se can enhance resistance to salt stress and mitigate the negative effects of salinity in plants. The underlying mechanisms are concluded as follows (Figure 3).

Se enhances photosynthesis. Se is effective in enhancing chlorophyll fluorescence parameters and photosynthetic pigment content and maintaining the ultrastructure of chloroplasts in plants under salt stress [99,100]. Diao et al. [101] found that Se can promote net photosynthetic rate and stomatal limitation and reduce intercellular CO_2_ concentration, thus promoting the photosynthesis of tomato seedlings under salt stress. Enhancement of photosynthesis may be because the chloroplast membrane system of leaf cells is less damaged and maintains better integrity by Se in plants under salt stress [102].

However, Se concentration must be set carefully because Se accumulated in high amounts in the leaves may inhibit the enzymatic kinetics or electron transport chain in photosynthesis [99,103,104]. The tertiary structure of most proteins depends on the formation of disulfide bonds (S-S). A new diselenide bond (Se-Se) or selenosulfide bond (Se-S) can easily form because of the replacement of Cys by SeCys in proteins when the plant cells contain a high concentration of Se [99]. This phenomenon disrupts the structure of the PSII complex in chloroplasts and exerts a strong inhibitory effect on photosynthetic electron transfer [99]. In addition, the substitution of Se for sulfur in the key enzymes involved in chlorophyll synthesis reduces their activity, disrupts the biochemical reaction, and severely hampers chlorophyll synthesis [99].

Se regulates osmotic pressure and maintains the stability of the plasma membrane in plants under salt stress. Se can increase the content of relevant osmotic protectants, such as proline, soluble sugars, and soluble proteins, and alleviate electrolyte leakage from the cell membranes in plants under salt stress [105]. Se also reduces the content of the lipid peroxidation product MDA and helps maintain membrane stability. For example, free proline content is increased by 72.7% and MDA content is reduced by 87.5% in NaCl-stressed wheat leaves treated with a low concentration (1–8 µmol L^−1^) of Na_2_SeO_3_. This treatment can also minimize electrolyte leakage from plant cell membranes by approximately 58% compared with the control group [99].

Se enhances antioxidant enzyme activity and alleviates oxidative damage. Se effectively enhances the activities of antioxidant enzymes (e.g., SOD, POD, APX, CAT), thus scavenging excess ROS and free radicals in the body and alleviating the oxidative stress damage caused by salt stress in plants. The enhancement of antioxidant enzyme activities and alleviation of oxdative stress in plants exposed to salt stress by Se have been reported in many species, such as wheat [99], bitter melon [106], soybean [107], melon [108], and peanut [109]. In melon plants, salt stress increases electrolyte leakage and MDA content. Treatment with Se can significantly reduce the symptoms of salt stress by significantly increasing the activities of SOD, POD, and CAT by 130%, 50%, and 20%, respectively, when compared with non-seleniferous melon plants, thus reducing the oxidative damage of ROS [108].

Se enhances the glyoxalase system to protect biomolecules, such as nucleic acids and proteins. Plants under salt stress accumulate excess methylglyoxal, which subsequently produces carbonyl stress, damaging the biomolecular proteins, DNA, RNA, lipids, and biofilms. The glyoxalase system in plants can effectively remove excess methylglyoxal while regulating GSH regeneration to maintain the dynamic balance in cells [110,111,112,113]. Rahman et al. [107] found that Se treatment enhances the antioxidant defense and glyoxalase systems of soybean under salt stress, thus protecting lipids, nucleic acids, proteins, and other biomolecules [105,106].

Se is effective in reducing Na^+^ uptake and accumulation in plants under NaCl stress. Increased salt concentrations in the soil decrease the ability of plants to take up water [87]. Once Na^+^ and Cl^-^ are taken up in enormous amounts by the roots, the metabolic processes would be impaired, and the photosynthetic efficiency would be decreased [86,114,115]. Sheikhalipour et al. [106] applied chitosan SeNPs to bitter melon under NaCl stress and found that this treatment increases the uptake of K^+^ but reduces the uptake of Na^+^, thus improving salt tolerance in bitter melon [116,117]. Similar results have been observed in maize and black bean. Interestingly, the uptake and translocation of Cl^-^ are not influenced by Se.

### 3.5. Se and Heavy Metal Stress

**Figure 4:**
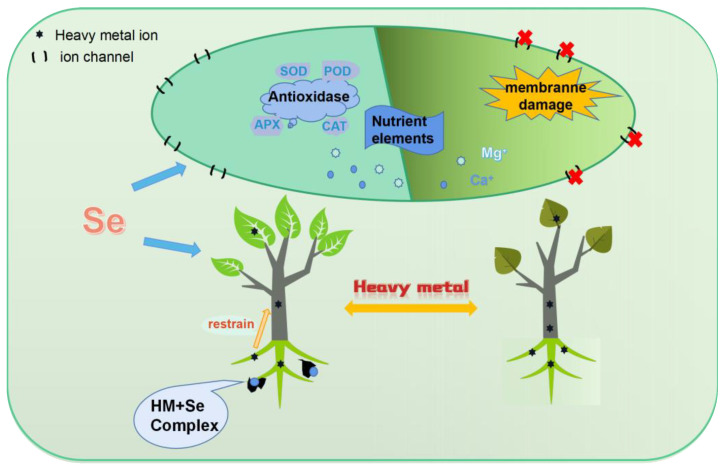
Patterns of Se mitigating heavy metal stress in plants. The left part shows Se treatment and the right is non-seleniferous treatment in plants under heavy metal stress.

Common heavy metals include Cd, Hg, Pb, Cu, and Cr, which are introduced into the ecosystem as a result of mining, chemical, and agricultural production activities. Heavy metals induce lipid peroxidation. The balance between the production and scavenging of free radicals in cells is disrupted when plants take up excessive amounts of heavy metals, resulting in the accumulation of large amounts of ROS, which further trigger the peroxidation of unsaturated fatty acids in membranes and damage membrane structure and function [118]. High concentrations of these elements not only cause poisoning in plants but also endanger the health of animals that consume them, eventually posing a risk to human health [119]. We review the studies concerning the correlation between Se and heavy metals and conclude the mechanism involved in the mitigation effects of Se on heavy metal stress in plants (Figure 4).

Se enhances plant photosynthesis. Heavy metal toxicity can inhibit photosynthesis by triggering the degradation of chlorophyll molecules by enhancing chlorophyllase activity and replacing the central Mg^+^ in the porphyrin ring, affecting overall plant growth and yield [120]. However, Se significantly enhances chlorophyll a, chlorophyll b, and total chlorophyll contents in plants under heavy metal stress, thereby facilitating photosynthesis [74,121]. Se also helps rebuild damaged cell membranes, chloroplast structures, and photosynthetic system components in plants [102,122,123,124].

Se enhances transpiration in plants. The water potential, leaf osmotic potential, and relative water content are significantly reduced in plants under heavy metal stress [74] because ion channels and stomata of cell membranes are damaged or even closed, resulting in the reduced transpiration and disruption of plant growth and metabolism [125]. Se can enhance transpiration and water transport in plants under heavy metal stress and reduce leaf temperature. For example, Cd stress inhibits the stomatal size, density, and stomatal conductance of *C. sativum* cells. A low concentration of SeNPs (126.6 µmol L^−1^) effectively improves the water potential (80%), leaf osmotic potential (52%), gas exchange properties, and transpiration rate of *C. sativum* [74].

Se enhances osmotic protection. It can enhance the proline and soluble sugar contents and help maintain the stability of the plasma membrane in plants under heavy metal stress [74]. Proline enhances membrane stability and reduces the degradation of proteins and carbohydrates [126]. Soluble sugars are important osmotic protectants for plants [127]. Application of low concentrations of SeNPs (126.6 µmol L^−1^) effectively enhances the proline content and total soluble sugar content of *C. sativum* under Cd stress by 39% and 64% [74], respectively, compared with those without Se treatment, thereby reducing the metal toxicity of Cd. Sun [128] demonstrated that Se application can reduce Cd-induced phytotoxic effects on cucumber plants by regulating stress response-related proteins and pathways, such as glycolysis pathway and nitrate assimilation pathway, which may increase Cd tolerance.

Se reduces the uptake and translocation of heavy metals in plants. Se promotes the formation of Fe plaques around the roots of plants under Hg stress [129], thus hindering heavy metals from entering the roots and accumulating in plants. Zhou and Li found that Se increases the adsorption of Hg2^+^ by Fe plaques in rice and causes an average of 1.42-fold increase in Se-treated plants compared with non-seleniferous plants [130]. Moreover, Se interacts with heavy metals. Thus, Se application leads to the formation of inert HgSe or/and HgSe-containing proteinaceous complexes in the rhizosphere [121]. For instance, Se forms insoluble HgSe precipitates with Hg in the roots, thus reducing the mobility and availability of Hg [129]. Similarly, Se reduces the levels of methylmercury (MeHg), a carcinogen in rice. The reduction in soil MeHg concentrations could be mainly attributed to the formation of Hg-Se complexes and thus reduction of microbial MeHg production [131]. Se can inhibit the translocation of heavy metals. The addition of selenite can significantly decrease the Cd concentrations in xylem sap, suggesting that Se can reduce Cd levels in the rice shoots by inhibiting Cd translocation from the roots to the shoots. This phenomenon could be ascribed to the increasing formation of Cd-thiol complexes in the roots and the reduction of Cd transport to the shoots [132].

Se can inhibit the translocation of heavy metals [132] because Se promotes the formation of heavy metal-thiol complexes, thereby reducing heavy metals in the xylem sap and the transfer of heavy metals from the roots to the shoots [133]. The common thiol compounds in plants include GSH, metallothionein, and phytochelatin (PCs) [134,135]. The complexes formed by PCs and heavy metals can be transferred into the vacuoles via vesicular membrane ATPases [136]. Therefore, the heavy metals can be segregated in the vacuoles [113].

Se enhances the uptake of nutrient elements, such as Ca, K, and Mg. Heavy metal stress can restrict the uptake and transfer of nutrient elements by plants and interfere with normal physiological activities, thus leading to the malnutrition of plants [74]. Se can not only inhibit the uptake of Cd but also mitigate the inhibitory effect of Cd stress on the uptake of nutrient elements in plants [137]. Water spinach [138] *Brassica napus* L. [14] and *C. sativum* [74] show an increase in the uptake of some essential elements, such as Ca, K, and Mg, but a reduction in the accumulation of Cd and Pb.

Se also regulates the expression of genes related to antioxidant enzymes. In mustard, Se affects the expression of genes encoding antioxidant enzymes (SOD, POD, CAT, GR, and GST-1) in mustard and increases the concentrations of some components in the cell wall (such as pectin, lignin, and hemicelluloses) [139,140]. It increases the thickness of the cell wall by regulating the expression of genes related to lignin synthesis (OsPAL, OsCoMT, and Os4CL3) [141], thereby stimulating the accumulation of metal(loid)s in the cell wall and inhibiting the transfer of heavy metals in plants [142,143].

Se increases the activity of antioxidant enzymes (e.g., SOD, POD, and GPX) and accelerates the removal of ROS induced by heavy metals in plants [42,144]. Heavy metal stress increases the synthesis of ROS and free radicals in plant tissues, leading to oxidative damage in plants. Treatment with SeNPs (126.6 µmol L^−1^) increases CAT activity by 76% compared with the control in C. sativum under Cd stress [74]. Therefore, Se decreases ROS (e.g., OH^−^, O^2−^, and H_2_O_2_) levels, which protects the plants from oxidative stress induced by heavy metals [145].

Se, although not essential for higher plants, can mitigate the damage of heavy metal stress. Se fertilizers can be used to regulate heavy metal stress in plants [146]. However, high concentrations of Se are toxic to plants because of changes in protein structure and function, particularly in Se-sensitive plants [9]. For example, improper concentrations of selenite increase lipid peroxidation and hydrogen peroxide production in *Brassica napus* L. under Cd stress, leading to further depletion of antioxidants and ultimately a further reduction in the biomass of the edible fraction of *Brassica napus* L. [147]. Therefore, the application of Se to alleviate heavy metal stress in plants must be carefully considered before it can be used in practical production.

## 4. Conclusions and Future Prospects

Abiotic stresses are abiotic environmental conditions that are not conducive to plant survival, growth, and development and even lead to injury, destruction, and death. Se can reduce the harmful effects of abiotic stresses on plants. This review describes the transportation and metabolism of Se in plants and summarizes the mechanisms by which Se alleviates abiotic stress in plants. This article concluded the functions of Se in abiotic stress in plants from several aspects. First, Se enhances the photosynthetic properties of plants. it can promote the synthesis of photosynthetic pigments and repair the damage of photosynthetic organelles, such as chloroplasts in plants. Second, Se enhances the activities of antioxidant enzymes. Se can significantly enhance the activities of antioxidant enzymes, such as SOD, POD, and CAT, and accelerate the removal of excessive ROS in plants. Third, Se regulates cell permeability and promotes the accumulation of resistant substances. Se can promote the content of osmoprotectants, such as proline and soluble sugar, thus maintaining cell osmotic pressure. Fourth, Se inhibits the transport of salt and metal ions in plants. Se can encourage these ions to form complexes or precipitate, effectively reducing their uptake in plants. Fifth, Se regulates the expression of related genes and proteins to improve plant tolerance. However, the specific mechanisms of these effects remain unclear.

Further investigations are needed to reveal the molecular mechanisms by which Se regulates abiotic stress response in plants. Some advanced technologies, such as the genomics, transcriptomics, and proteomics, can help identify the key genes, metabolites, proteins, and regulators that play key roles in the regulation of Se on abiotic stress. The detailed functions of these key genes, proteins, and metabolites can be characterized using genetic engineering approaches. Thus, some abiotic stress-tolerant species would be created and reserved as germplasm resources. These germplasm resources may play roles in the future. Applying suitable concentrations of Se in crops may be a selectable way to mitigate crop losses in the presence of abiotic stress. However, the application of Se needs caution because inappropriate concentrations of Se may exert toxic effects on plants.

## Data Availability

Not applicable.
